# Development, Characterisation and High-Temperature Suitability of Thin-Film Strain Gauges Directly Deposited with a New Sputter Coating System

**DOI:** 10.3390/s20113294

**Published:** 2020-06-10

**Authors:** Daniel Klaas, Rico Ottermann, Folke Dencker, Marc Christopher Wurz

**Affiliations:** Institute of Micro Production Technology, Leibniz Universität Hannover, An der Universität 2, 30823 Garbsen, Germany; dencker@impt.uni-hannover.de (F.D.); wurz@impt.uni-hannover.de (M.C.W.)

**Keywords:** direct deposition, sputtering, sensors, micro strain gauges, temperature coefficient of resistance, gauge factor, k-factor, quarter-bridge, half-bridge, trimming, high-temperature

## Abstract

New sensor and sensor manufacturing technologies are identified as a key factor for a successful digitalisation and are therefore economically important for manufacturers and industry. To address various requirements, a new sputter coating system has been invented at the Institute of Micro Production Technology. It enables the deposition of sensor systems directly onto technical surfaces. Compared to commercially available systems, it has no spatial limitations concerning the maximum coatable component size. Moreover, it enables a simultaneous structuring of deposited layers. Within this paper, characterisation techniques, results and challenges concerning directly deposited thin film strain gauges with the new sputter coating system are presented. Constantan (CuNiMn 54/45/1) and NiCr 80/20 are used as sensor materials. The initial resistance, temperature coefficient of resistance and gauge factor/k-factor of quarter-bridge strain gauges are characterised. The influence of a protective layer on sensor behaviour and layer adhesion is investigated as well. Moreover, the temperature compensation quality of directly deposited half-bridge strain gauges is evaluated, optimised with an external trimming technology and benchmarked against commercial strain gauges. Finally, the suitability for high-temperature strain measurement is investigated. Results show a maximum operation temperature of at least 400 °C, which is above the current state-of-the-art of commercial foil-based metal strain gauges.

## 1. Introduction

Strain gauges are used as universal measuring devices for detecting mechanical strain within technical components. Electrical strain gauges can be divided into semiconductor and commonly used metal strain gauges [1]. Usually, a metal strain gauge consists of at least one meander-shaped sensor structure on a thin carrier substrate, for example a few ten micrometre thin polyimide foil, contact pads and a protecting top layer [2]. Such strain gauges are glued onto the surface of a component. The working principle can be roughly described as follows: When a strain is applied, the component deforms causing a mechanical elongation of the meander-shaped sensor structure that results in a change of resistance. The resistance change can be measured and transformed into actual strain. The temperature behaviour of such strain gauges is described by the temperature coefficient of resistance (TCR) (Equation (1)). The elongation sensitivity is expressed by the gauge factor, in the following called the k-factor (Equation (2)). Unfortunately, adhesives and carrier substrates are causing measurement deviations (for instance: zero drift and reduction of sensitivity) as a result of ageing phenomena such as relaxation and creep [3,4]. Nowadays, especially in the context of Industry 4.0, demands on compact sensor systems usable in space limited positions or positions with high operation temperatures within technical systems are increasing rapidly [5]. Commercially available substrate-based strain gauges appear inappropriate.

One very promising technology is the usage of strain gauges directly deposited onto technical surfaces of components. As a result, no carrier substrate or glue is required resulting in a thinner sensor. Depending on the electrical conductivity of the surface, an insulation layer might be essential. The sensor layout can be adapted to the specific application and electrical supply lines can be deposited individually. Moreover, sensor and insulation layer materials can be chosen individually, enabling an application-specific sensor useable at high temperatures of, theoretically, up to a few hundred degrees Celsius. For the manufacturing of strain gauges and directly deposited strain gauges, chemical vapour deposition and physical vapour deposition technologies are used, mainly sputtering [6,7,8]. Structuring is done using shadow masks, photolithography or laser ablation [8,9,10,11]. As sensor layer, a variety of materials is used. The most common sensor materials are Constantan (CuNiMn 53–57/43–45/0.5–1.2) and NiCr [2]. Directly deposited strain gauges are manufactured in commercial vacuum coating systems [8,12]. Such systems feature a vacuum chamber of limited size resulting in restrictions concerning the maximum dimensions of the coatable components. To overcome these disadvantages, a new sputter coating system has been invented. It enables a simultaneous deposition and structuring of insulation, sensor and protective layers on components of arbitrary size. The system is usable outside a clean room and directly integrable in a production line. Further information about the system and the deposition sequence are already published in detail [13,14,15]. The system setup and manufacturing features, where the component to be coated becomes one part of the chamber, is related to pending [16,17] and granted patents [18].

## 2. Manufacturing and Contacting of Strain Gauges

Manufacturing of directly deposited strain gauges can be roughly divided in four steps. These are: substrate preparation, insulation layer deposition, sensor layer deposition and contacting. For validation of sensors and in order to detect the main challenges, test depositions of thin-film strain gauges on AlMg3-substrates (3.3535) were conducted.

### 2.1. Substrate Preparation

For depositing sensors, measurement results show that a polishing of the substrate is crucial to reduce the surface roughness and thereby reduce the required layer thickness for sufficient electrical insulation properties [14,19]. To enable the deposition of sensors on various technical components, common polishing techniques like chemical-mechanical-polishing are not suitable. Therefore, a commercial Dremel^®^4000 multitool is used with Wolfcraft^®^ 2134000 2/B polishing paste. In addition, different polishing head versions have been tested. 

Best results are achieved by a ten minute manual polishing with a Hoffmann Group^®^ Garant 552100ZY1012 polishing head at an angle of approximately 45° between the component and the multitool at 20,000 rpm and a contact pressure of approximately 1.1 ± 0.1 MPa. A surface roughness tester HOMMEL-ETAMIC W5 from JENOPTIK is used for checking the mean roughness depth R_z_ and the average roughness R_a_ within a polishing area of approximately 30 × 30 mm with a measuring length of 4.8 mm and a measurement tip radius of 2 µm. The mean roughness depth R_z_ is reduced from 1.43 µm of the untreated surface to 0.57 µm and the average roughness R_a_ from 0.27 µm to 0.1 µm. Before a subsequent deposition, cleaning of substrates with acetone and isopropanol is necessary in order to remove organic residues of the polishing paste.

### 2.2. Layer Deposition

**Figure 1:**
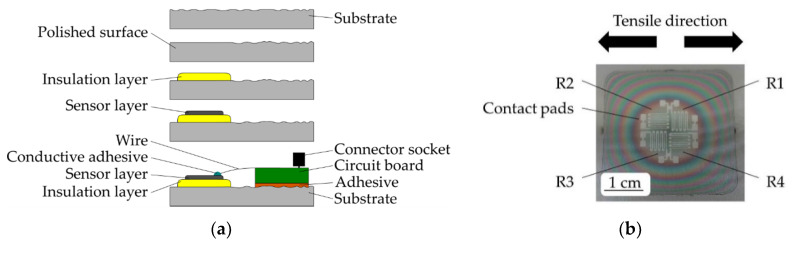
(**a**) Manufacturing steps and contacting of directly deposited strain gauges. (**b**) Four strain gauges after deposition. The quarter-bridge strain gauges are named R1 to R4 and connected to two half-bridge strain gauges. Arrows indicate tensile direction during characterisation.

**Figure 2:**
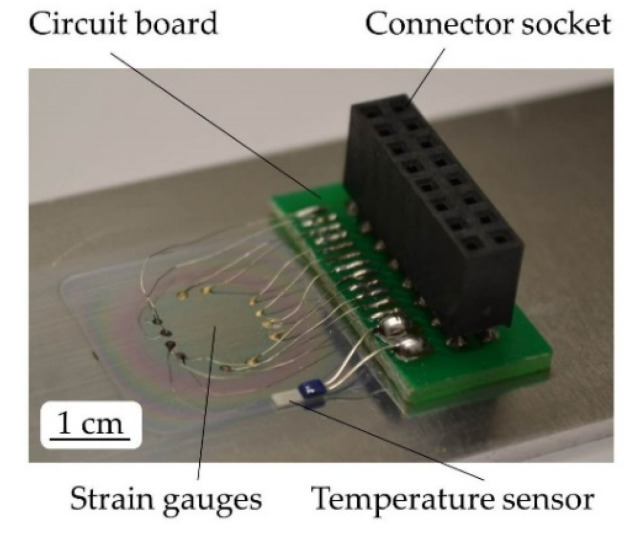
Directly deposited strain gauges after contacting.

In the following, a thin Al_2_O_3_ insulation layer is sputter deposited in an area of 20 × 20 mm on the substrate. As a result of the manufacturing process, the maximum layer thickness of approximately 1 µm decreases laterally. The insulation layer (compare [14]) is deposited by using a power of 275 W, an argon flux of 15 sccm and a sputter pressure of approximately 9.2 × 10^−3^ mbar. On top of the insulation layer, a structured sensor layer of Constantan (CuNiMn 54/45/1) or NiCr 80/20 is deposited and simultaneously structured using silicon shadow masks manufactured by means of deep-reactive ion beam etching [15]. Manufacturing of Constantan and NiCr 80/20 layers takes place with a power of 200 W, an argon flux of 7.5 sccm and a sputter pressure of approximately 6.8 × 10^−3^ mbar. Both sensor materials are chosen as they enable a low TCR that simplifies temperature compensation and features a sufficient k-factor. Strain gauges are connected to a circuit board by nickel-plated copper wires. The circuit board is glued onto the technical surfaces and includes a connector socket (compare Figure 1a and Figure 2).

### 2.3. Contacting

In order to prevent an influence of electrical supply lines or contact resistances, a four-wire contacting technology is used to validate the strain gauges. An isotropic conductive adhesive, Chemtronics^®^ CW 2400, connects the sensors to the copper wires electrically.

Temperature tests show a low increase in resistance of less than 0.6 Ω for the conductive adhesive in a temperature range of up to 400 °C. Manufacturing steps and contacting are illustrated in Figure 1a. The sensor layout before contacting is shown in Figure 1b, after contacting in Figure 2.

One shadow mask includes four meander-shaped quarter-bridge strain gauges, consisting of nine lines with a width of approximately 120 µm each, forming two half-bridge strain gauges arranged as shown in Figure 1b. The thickness of the sensor layer equals approximately 350 nm for Constantan and 500 nm for NiCr 80/20. Deposition takes place on AlMg3-tensile specimens. DIN 50125 Type H was partially considered for the geometry design (300 × 40 × 2 mm) of tensile specimens [20].

## 3. Characterisation

Characterisation of strain gauges is realised with respect to VDI Directive 2635 [21]. They are characterised concerning initial resistance, TCR and k-factor. For evaluation, a QuantumX MX1615B strain gauge bridge-amplifier from Hottinger Baldwin Messtechnik GmbH (HBM) is used.

### 3.1. Parameters

#### 3.1.1. The Initial Resistance

*R_0_* describes the initial resistance and is measured directly after deposition at room temperature of 21 ± 2 °C under tensile-free conditions. Sensors are named R1-R4 as shown in Figure 1b.

#### 3.1.2. The Temperature Coefficient of Resistance

The *TCR* is defined by Equation (1) and describes the relative resistance change with respect to change in temperature. *ΔR* represents the resistance change, *R_0_* the initial resistance and *ΔT* the temperature change [22]. The lower the *TCR*, the smaller is the temperature influence on the resistance value:

(1) $$TCR=\frac{\Delta R/R_{0}}{\Delta T}$$

#### 3.1.3. The K-Factor

The k-factor describes the elongation sensitivity of the strain gauge and is described by Equation (2), where *∆R* equals the resistance change, *R_0_* the initial resistance and *ε* the elongation of the specimen [4]:

(2) $$k=\frac{\Delta R/R_{0}}{\varepsilon }$$

The k-factor is evaluated using a Mecmesin MultiTest 2.5-xt commercial tensile test machine. By stretching the specimen with a tensile force, the elongation of the specimen causes a measureable change of the strain gauge resistance. The elongation is calculated from tensile force *F*, cross-sectional area *w* × *d* and previously measured temperature-dependent Young’s modulus *E(T)* of the tensile specimen (Equation (3)).

(3) $$\varepsilon =\frac{F}{w\times d\times E\left(T\right)}$$

The k-factor is finally calculated using Equation (4), which includes the initial resistance *R*_0_, the resistance change *∆R*, the temperature-dependent Young’s modulus *E(T)*, the actual temperature *T*, the room temperature before the measurement *T_room_*, the maximum tensile force *F_max_*, the pre-force *F_pre_*, as well as the temperature-dependent geometry of the specimen described by the cross-sectional area *w* × *d* of the specimen and the thermal elongation coefficient *α*.

(4) $$k\left(T\right)=\frac{\frac{\Delta R\left(T\right)}{R_{0}\left(T\right)}\times E\left(T\right)\times \frac{1}{F_{max}-F_{pre}}}{{\left(w\times d\times {\left(1+\alpha \times \left(T-T_{room}\right)\right)}^{2}\right)}^{-1}}$$

#### 3.1.4. The Layer Adhesion

To enable a high-quality strain measurement, a sufficient layer adhesion between the substrate and insulation layer, as well as between insulation layer and sensor layer, is required. For evaluating the layer adhesion, an adhesive tensile testing method related to EN ISO 4624:2016 is used. A tension stamp with a defined diameter is glued with Loctite^®^ 401 onto the deposited layers. The tensile test takes place using a Mecmesin MultiTest 2.5-xt tensile test machine. Tensile force is applied perpendicular to the substrate surface. During testing, tensile force is continuously increased. The maximum tensile force *F_maxT_* is reached as soon as the tension stamp is separated from the substrate surface. The layer adhesion *σ* is calculated according to Equation (5), where *r* equals the radius of the tension stamp:

(5) $$\sigma =\frac{F_{maxT}}{\pi \cdot r^{2}}$$

### 3.2. Results

Firstly, Constantan strain gauges are deposited and evaluated. They are marked c1-c4 and consist of four quarter-bridges, R1-R4, each. Investigations show that differences between TCR values and k-factors at room temperature can be reduced by a regular cleaning of the system. A manufacturing in batches with a maximum of five to six depositions is recommended. After every batch, a sandblasting of the shadow mask carrier (the one that is used for the insulation layer deposition), chimney and gas injection plate of the sputter source is carried out in combination with a cleaning of the process chamber. Deposition of NiCr 80/20 on samples n1-n4 takes place in one batch after a cleaning. Eight such samples, with four quarter-bridges, R1-R4, each, are used for evaluating the initial resistance, TCR and k-factor temperature dependency of NiCr 80/20.

#### 3.2.1. The Initial Resistance

**Figure 3:**
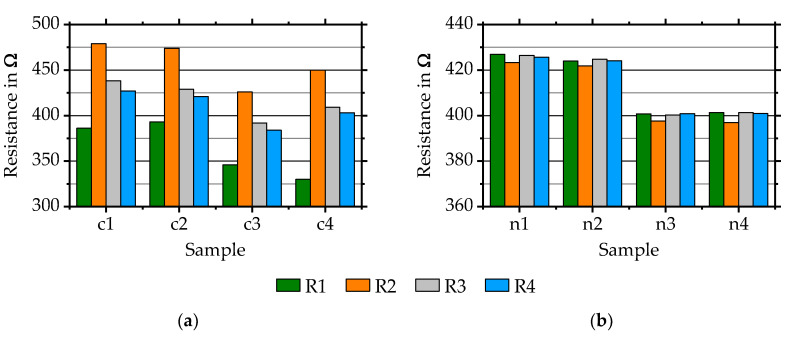
Initial resistances R1–R4 of (**a**) four Constantan samples c1–c4 and (**b**) four NiCr 80/20 samples n1–n4 deposited after each other.

Figure 3a shows four samples of Constantan deposited by using shadow masks manufactured related to [15]. Each sample is measured directly after deposition. Results show variances of up to 44% between all resistance values and up to 36% within simultaneously deposited strain gauges. Reasons for this are mainly related to the shadow mask manufacturing process. Improvements are achieved by using a cleaned deep-reactive ion etching system as used for manufacturing the shadow masks for NiCr 80/20 depositions (Figure 3b).

NiCr 80/20 samples are deposited after each other onto AlMg3-substrates. Resistance values within simultaneously deposited quarter-bridge strain gauges R1–R4 of each sample are homogeneous and vary up to 1.7%. Maximum differences between all single resistance values equals 7.6%. Most likely, differences within initial resistances of simultaneously deposited quarter-bridge strain gauges of one sample are caused by: various widths of shadow mask structures, the gap between the mask and substrate, substrate surface roughness and layer homogeneity influenced by shadow mask carrier geometry. Differences between initial resistances of various samples might be a result of the gap between mask and substrate, surface roughness and variable layer thicknesses caused by for instance a variable sputter power. So far, no influence of sensor material on the achievable homogeneity of initial resistances values has been detected. More detailed investigations are carried out at the moment.

#### 3.2.2. The Temperature Coefficient of Resistance

For evaluating the TCR, substrates with deposited and contacted sensors on their surface are heated from room temperature (21 ± 2 °C) to slightly above 100 °C on a heating plate. Temperature measurement takes place with an additional type PTFM101B1A0 platinum temperature sensor from TE Connectivity attached to the substrate surface (compare Figure 2). 

**Figure 4:**
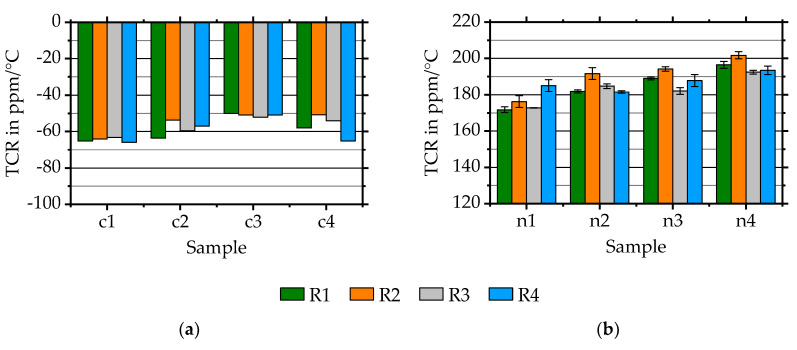
*TCR* values of (**a**) directly deposited Constantan samples and (**b**) of NiCr 80/20 samples.

During cooling to room temperature, the resistance change is measured by using the strain gauge bridge-amplifier. In literature, the value depends on the layer thickness and substrate material. Measurement results are shown in Figure 4. 

The average *TCR* is evaluated to −58 ± 6 ppm/°C for Constantan and to 186 ± 8 ppm/°C for NiCr 80/20. The *TCR* values of Constantan samples are varying with a standard deviation of approximately 10%. Although the samples of NiCr 80/20 are deposited after each other within a cleaned system, *TCR* values are changing slightly between samples. Within one sample the change is 1.8% with a maximum of 2.9% showing high reproducibility. The standard deviation for all samples is approximately just 4%.

However, both *TCR* values compare well to literature values. For Constantan values of ± 40 ppm/°C for bulk material [23] and for instance −70 ppm/°C for 400 nm thin Constantan layers on oxidised silicon substrates with a metallic adhesion layer [24] are listed. The NiCr 80/20 literature values range from 85 ppm/°C for bulk NiCr 80/20 [25] to for instance 300 ppm/°C for 300 nm thin NiCr 80/20 on an insulated nickel-based superalloy [26].

#### 3.2.3. The K-Factor at Room Temperature

For characterising Constantan samples, a tensile force of 50 N is used. For NiCr 80/20 samples, the measurement setup is improved by using a higher maximum load of 2000 N with an included pre-force of 500 N. The higher maximum force reduces the standard deviation of the k-factor measurement. The pre-load is used to avoid a sliding of the specimen within the clamping jaws.

**Figure 5:**
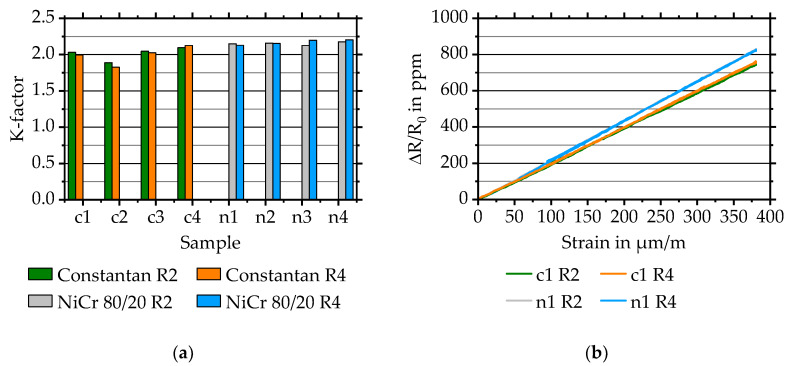
(**a**) Evaluated k-factors for directly deposited Constantan (c1–c4) and NiCr 80/20 (n1–n4) quarter-bridge strain gauges R2 and R4. (**b**) Relative resistance change *ΔR/R_0_* over strain *ε* for two simultaneously deposited quarter-bridges, R2 and R4, made of Constantan (c1) and NiCr 80/20 (n1).

The k-factor at room temperature (21 ± 2 °C) is calculated for the quarter-bridge strain gauges R2 and R4 of each specimen as they are orientated in tensile direction. Measurement results are shown in Figure 5. The average k-factor equals 2.00 ± 0.09 for Constantan and 2.16 ± 0.03 for NiCr 80/20, corresponding to Figure 5a. 

These values correlate with literature values of 2.0 for Constantan and 2.1 to 2.5 for NiCr 80/20 that are depending on for instance substrate material [4]. 

The relative resistance change *ΔR/R_0_* over strain *ε* is shown in Figure 5b, proving the linear sensor behaviour for both sensor layer materials. All sensors made of Constantan and NiCr 80/20 show a linear behaviour. For an better overview, just the resistance values of two quarter-bridges of two simultaneously manufactured half-bridges are shown.

#### 3.2.4. The Layer Adhesion at Room Temperature

Layer adhesion *σ* is evaluated on 3.3535 AlMg3-substrates for sensors made of 350 nm Constantan and 500 nm NiCr 80/20 deposited onto Al_2_O_3_ insulation layers. With respect to Section 3.1.4, adhesion tests take place with and without Al_2_O_3_ protective layers deposited above the sensor layer. Measurements show a delamination between the insulation layer and AlMg3-substrate independently of sensor layer material and existence of protective layer. The achieved layer adhesion *σ* equals 9.6 ± 3.1 MPa. The average value is twice as high as the value of 4.8 ± 0.4 MPa achieved with commercial foil-based strain gauges attached with Hottinger Baldwin Messtechnik GmbH Z 70 glue to substrate surface.

#### 3.2.5. Influence of Protective Layer and Temperature Dependency of K-Factors

Measurement results show a slight increase of the resistance of Constantan samples over time already at room temperature and increasing with temperature. Such behaviour makes it difficult to evaluate the k-factor for Constantan over a temperature range of up to 100 °C. Reasons for this are expected to be related to oxidation.

**Figure 6:**
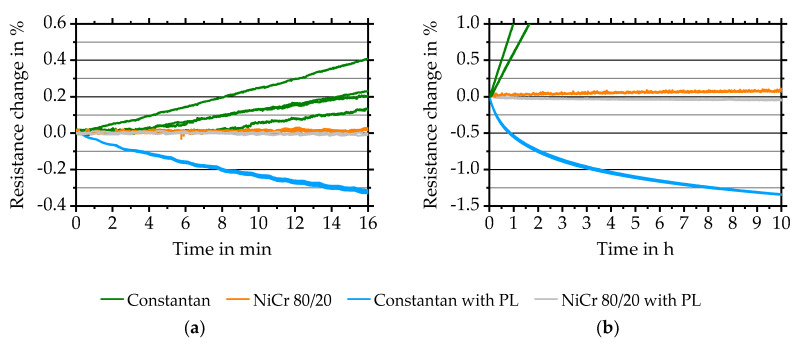
(**a**) The resistance change of Constantan and NiCr 80/20 quarter-bridges R1-R4 with protective layer (PL) compared to Constantan and NiCr 80/20 quarter-bridges without protective layer at 150 °C. (**b**) Ten hours long-time behaviour of Constantan and NiCr 80/20 quarter-bridges, R1 and R2, with and without protective layer at 150 °C.

To investigate the behaviour, a 1.3 µm thick Al_2_O_3_ insulation layer is deposited on top of the Constantan sensor layer. Layer deposition takes place with same parameters as used for the substrate-to-sensor insulation layer. Figure 6a shows results of the first 16 min for sensors made of Constantan and NiCr 80/20 with and without a protective layer.

Without the protective layer, Constantan changes more rapidly than NiCr 80/20, most likely as a result of the higher oxidation probability related to copper [27], whereas NiCr 80/20 shows passivating behaviour. Long-time measurements with protective layers show a decrease in resistance change over time for Constantan and a slightly increasing resistance change for NiCr 80/20 over time (Figure 6b). Reasons for this are most likely related to annealing effects and are under investigation.

**Figure 7:**
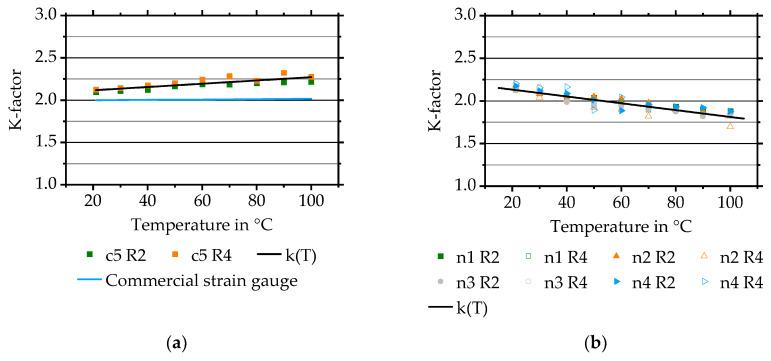
(**a**) K-factor temperature behaviour of Constantan quarter-bridges, manufactured on one sample, compared to a commercial strain gauge. (**b**) K-factor temperature behaviour of NiCr 80/20.

As a result, it is possible to reduce or, compared to the measurement time, rather avoid an increase of resistance at constant temperature by using the protective layer (sample c5). Measurements show that evaluation of k-factor over the required temperature range up to 100 °C for Constantan is possible (compare Figure 7a). The k-factor exhibits a linear behaviour with a gradient of 0.0021 1/°C resulting in a k-factor of 2.11 ± 0.02 at room temperature and 2.25 ± 0.03 at 100 °C.

Literature values for Constantan are depending on the stoichiometry of CuNiMn and are typically varying between 1.9 over 2 (CuNiMn 55/44/1) to 2.15 (CuNi 60/40) [4] with a relatively low temperature dependency of the k-factor with a typical value of 93 × 10^–6^ 1/°C [28]. Such a temperature dependency for a commercial strain gauge with a k-factor at room temperature of 2.0 is shown in Figure 7a.

Achieved values for directly deposited strain gauges with stoichiometry of CuNiMn 54/45/1 correspond to literature values at room temperature but show an approximately 8% higher temperature dependency with a value of 2.25 at 100 °C compared to just 2.09 for commercial ones. Reasons for this are not completely understood yet. They might be a result of the stoichiometry or the insulation layer [4] and are under investigation.

The k-factor temperature behaviour of NiCr 80/20 is shown in Figure 7b. The k-factor is reproducibly linearly decreasing for each sample from an average of 2.16 ± 0.03 at room temperature to an average of 1.83 ± 0.06 at 100 °C. This equals a reduction of approximately 15%. Commercial strain gauges made of NiCr 80/20 demonstrate a k-factor of 2.1–2.5 [4]. Temperature dependency for NiCr strain gauges from HBM Series KFU, with non-stated layer thickness, equals 4.5 × 10^–4^ 1/ °C [29] compared to 41.7 × 10^–4^ 1/ °C for the k-factor shown in Figure 7b.

For subsequent experiments, measurements are carried out exclusively with NiCr 80/20. The reason is that the configuration without a cover layer allows the usage of a unit to externally trim the initial resistances as it is required for enabling sufficient temperature compensation (compare to Section 4).

## 4. Temperature Compensation Capability

To evaluate the temperature compensation capability, five half-bridge strain gauges are deposited on AlMg3-substrates. Theoretically, two identical quarter-bridge strain gauges connected in a half-bridge configuration (like R1 with R2 and R3 with R4) enable complete temperature compensation [1]. The half-bridge strain gauges are heated to 100 °C without applying any strain. As a result, the sensors are expected to measure an apparent strain of 0 µm/m. The strain is calculated according to Equation (6), where *ν* represents the Poisson’s ratio, *U_S_* the supply voltage, *U_O_* the output bridge voltage and *k(T)* the individually evaluated temperature-dependent k-factor:

(6) $$\varepsilon =\frac{4}{k\left(T\right)}\cdot \frac{1}{1+\nu }\cdot \frac{U_{O}}{U_{S}}$$

The average apparent strain of five half-bridge strain gauges equals 164.6 µm/m with a maximum apparent strain of 344 µm/m. To validate the apparent strain for commercially available strain gauges, measurements with commercial half-bridge strain gauges are carried out. The half-bridges are glued on AlMg3-substrates. Evaluation is carried out as described above. The result is an apparent strain of 13.9 ± 8.8 µm/m with a maximum of 26 µm/m.

**Figure 8:**
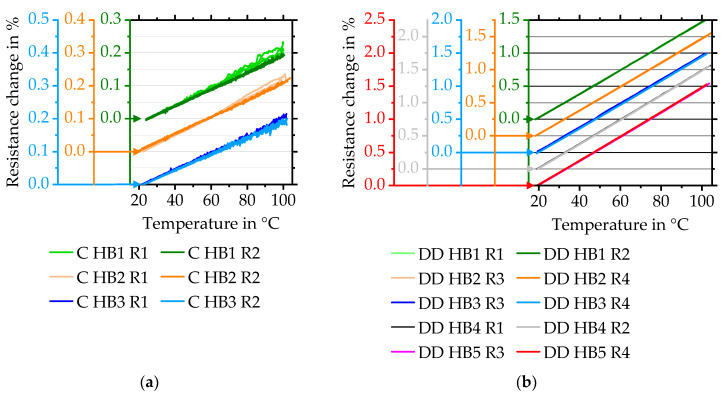
Temperature behaviour of two resistances forming one half-bridge. Curves are evaluated after cooling down from 100 °C to 25 °C. Results are shown for (**a**) commercial and (**b**) directly deposited strain gauges. Abbreviation C means commercial; DD stands for directly deposited.

Corresponding to the literature [22], reasons for the high apparent strain of directly deposited strain gauges are primarily related to various initial resistances or various temperature dependencies of two quarter-bridges forming a half-bridge. To determine the actual variances and possible tolerances, commercial strain gauges are evaluated concerning their resistance behaviour in dependence of temperature and their initial resistances and are benchmarked against directly deposited ones. Three temperature curves of single quarter-bridges during cooling down to room temperature for three commercial half-bridges and five directly deposited strain gauges are shown in Figure 8. Each gradient is listed in Table 1. Compared to commercial strain gauges (Figure 8a), the curves of resistance change of directly deposited sensors show higher reproducibility and linearity as shown in Figure 8b. The linearity is described by the gradient of linear regression that can be interpreted as the individual *TCR* of each strain gauge (compare Table 1).

**Figure 9:**
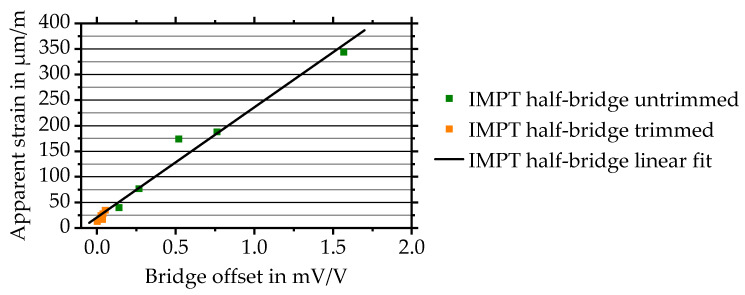
Apparent strain of trimmed and untrimmed half-bridges. Fit is generated from all shown half-bridge values.

Both directly deposited quarter-bridge strain gauges forming one half-bridge show nearly identical temperature behaviour. Commercial sensors demonstrate a higher average TCR difference of 8.0 ± 3.2% compared to just 1.0 ± 0.8%. This might be related to adhesive layer and surface preparation influences [4]. Average and standard deviation of the TCR equals 181.3 ± 1.7 ppm/°C for directly deposited compared to lower values of just 26.0 ± 1.5 ppm/°C for commercial strain gauges. To reduce the resulting apparent strain, trimming is used. In general, trimming is required due to various reasons: Equal sensors are expected to show equal behaviour. Moreover, trimming is crucial for the use of common equations, such as Equation (6), as it is based on assumptions that both resistances have nearly identical values [4]. As a result and due to design layouts, commonly available bridge-amplifiers are limited by a maximum bridge-offset. Such value equals ±5 mV/V for the HBM QuantumX MX1615B bridge-amplifier used within this paper, for instance. To enable trimming, an external trimming technology is developed. It consists of a rotating polishing head that is lowerable by a piezoelectric motor onto a quarter-bridge. It allows decreasing the thickness of strain gauges in order to equalise the initial resistances and to trim the bridge configuration. All five directly deposited half-bridges are trimmed at room temperature of 21 ± 2 °C to a bridge voltage close to 0 mV/V equalling a resistance difference of 0%. Afterwards, sensors are heated from room temperature to 100 °C for three times. The measured average apparent strain of the trimmed directly deposited strain gauges is depicted in Figure 9 and compared to untrimmed strain gauges. A linear fit is generated from all half-bridge values and shown additionally in Figure 9.

As a result of trimming, the remaining average apparent strain is reduced by 86% to 23.4 ± 7.8 µm/m with a maximum apparent strain of 35 µm/m. This is much closer to values of the apparent strain for commercial sensors of 13.9 ± 8.8 µm/m with a maximum of 26 µm/m. Taking into account that a tensile force of 2000 N is causing a strain of approximately 350 µm/m, trimming appears essential to reduce the apparent strain. More importantly, it directly influences the achievable resolution within the temperature range.

## 5. High-Temperature Suitability

### 5.1. Sensor Behaviour

**Figure 10:**
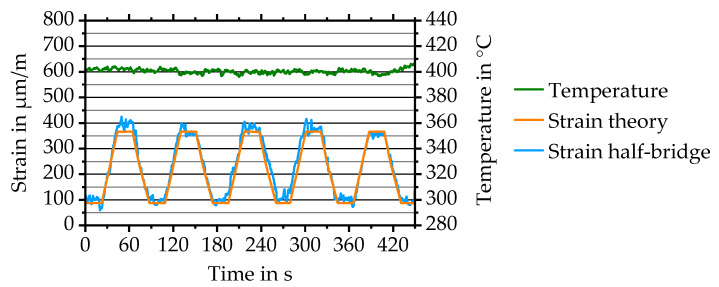
Theoretical strain and measured strain of a directly deposited half-bridge at cyclical loading between 500 and 2000 N at 400 °C.

Through trimming, the apparent strain is significantly reduced allowing to evaluate the actual sensor behaviour. Maximum operation temperature above 350 °C, the state-of-the-art of commercial foil-based sensors [4], is of special interest. To achieve higher operation temperatures, the sensor setup, substrate and contacting are adjusted. To improve the layer adhesion on Al_2_O_3_ insulation layer, a chromium adhesion layer is used. The revised sensor stack consists of 1.8 µm Al_2_O_3_ insulation layer, 20 nm Chromium, 500 nm NiCr 80/20, 20 nm Chromium, 1.4 µm Al_2_O_3_ protective layer. Temperature treatment shows positive influence on sensor behaviour (compare Figure 6b). Therefore, a four hours thermal treatment at 500 °C is carried out. To reduce potential carrier substrate influences on the maximum operation temperature, 1.4301 stainless steel with a geometry of 300 × 40 × 0.8 mm³ is used. Al_2_O_3_ contact boards replace circuit boards. Boards are attached to the steel substrate by high-temperature silicone and electrical wires by conductive adhesive. A Mecmesin MultiTest 2.5-xt commercial tensile test machine is used for applying force and a closed-loop controlled heating plate for applying temperature. Results for a cyclical load with a frequency of 0.012 Hz (period duration 85 s) with a tensile pre-force of 500 N and a maximum tensile force of 2000 N are evaluated and detailed in Figure 10.

The maximum temperature reached equals 400 °C. Strain is calculated from Young’s modulus and force corresponding to Equation (4). Measurement results show maximum operation temperature 50 °C higher than the one of commercial foil-based strain gauges. The measured average strain at 2000 N equals approximately 374.6 ± 17.4 µm/m with a maximum of 400 µm/m compared to the theoretical value of 366 µm/m. Approximately 22% of the measured cycles, as shown for cycle 5 in Figure 10, show outstanding accordance between the theoretical and measured strain courses with a maximum deviation of just 32 µm/m. Corresponding to the deviations, the best minimum force resolution equals approximately 175 N. The maximum temperature is limited due to liquefaction of solder connection of the temperature sensor and separation of the high temperature silicone resulting in detached Al_2_O_3_ contact boards. Trimming is not possible for strain gauges with protective layer, resulting in half-bridge offsets of −3.05 and −2.82 mV/V. The k-factor is evaluated to 1.90 ± 0.07 at 400 °C compared to 1.91 ± 0.14 at room temperature.

### 5.2. Layer Adhesion after High-Temperature Treatment

To proof layer adhesion *σ*, adhesion tensile tests have been carried out on steel substrates as described in Section 3.1.4. The layer delamination occurs between insulation layer and substrate material as already detected for AlMg3-substrates. Before temperature treatment, the layer adhesion *σ* on steel equals 17.3 ± 1.5 MPa, showing outstanding layer adhesion properties compared to 12.5 ± 2.5 MPa for commercial foil-based strain gauges attached with HBM Z 70 glue. After a temperature treatment of directly deposited strain gauges at 400 °C for 2 h, *σ* equals 17.1 ± 3.0 MPa. Instead of an expected decrease of layer adhesion, the adhesion remains stable with an extended standard deviation. Further research has to be carried out to evaluate the long-term layer adhesion.

## 6. Discussion, Summary and Outlook

A new sputter coating system has been developed and successfully used to manufacture directly deposited micro strain gauges made of Constantan and NiCr 80/20 on 3.3535 AlMg3 and 1.4301 stainless steel substrates. A characterisation technique and corresponding measurement setups have been invented with respect to the VDI-Directive 2635.

However, literature shows a variety of existing challenges and issues to investigate. Some of which are related to the new sputter coating system while others are more generally related to direct deposition that is also possible within commercial sputter devices.

The *TCR*, k-factor and k-factor temperature dependency have been evaluated for Constantan and NiCr 80/20 strain gauges. Using a new deep-reactive ion etched shadow mask structuring technology, achievable initial resistances are shown. *TCR* and k-factor reproducibility have been improved by a regular cleaning procedure per batch involving sputter source, shadow mask carrier and process chamber as well as manufacturing in batches with a maximum of five to six samples. The average k-factor at room temperature of 2.00 ± 0.09 (Constantan) and 2.16 ± 0.03 (NiCr 80/20) as well as the average TCR of −58 ± 6 ppm/°C (Constantan) and 186 ± 8 ppm/°C (NiCr 80/20) are largely corresponding to literature values. The k-factor temperature dependency of Constantan shows a gradient of 21.0 × 10^–4^ 1/°C and NiCr 80/20 of 41.7 × 10^–4^ 1/°C that are up to ten times higher as values for commercial sensors. However, a comparison to k-factor temperature dependencies of commercial strain gauges is possible only to a limited degree, as the listed values are depending on layer thickness that is often not stated [30]. Moreover, the result might be related to stoichiometry differences or residual stress. A final evaluation is not possible. Further research has to be conducted. Measurements of the k-factor temperature dependency of Constantan samples without a protective layer are not possible, as resistance already increases significantly at a constant temperature above room temperature.

The influence of protective layers has been investigated. Such layers have been proven to stabilise temperature and long-term behaviour of the sensor system. Reasons are related to annealing and oxidation. Directly deposited strain gauges have been compared to commercial strain gauges concerning *TCR*, apparent strain, layer adhesion and temperature compensation capability. Results show a lower *TCR* difference and more uniform TCR behaviour of two simultaneously manufactured quarter-bridges forming one half-bridge compared to commercial strain gauges. The temperature compensation capability has been tested using quarter-bridge strain gauges connected to half-bridge strain gauges showing high apparent strain. A reduction of apparent strain by approximately 86% to an average of 23.4 ± 7.8 µm/m (directly deposited) compared to 13.9 ± 8.8 µm/m (commercial) has been achieved through usage of an external trimming technology. Results demonstrate that *TCR* and initial resistance, as well as change of quarter-bridge resistance values over temperature and time, need to be as identical as possible to achieve well-performing sensors. Finally, tests for maximum achievable temperatures have been carried out. Directly deposited sensors show a 50 °C higher operation temperature compared to commercial state-of-the-art foil-based metal strain gauges. Their acceptable strain sensitivity equals 32 µm/m corresponding to a force of 175 N for the depicted sample geometry, which promises great potential for high-temperature applications currently limited only by the contacting technology. Investigations of the layer adhesion on steel show values of 17.3 ± 1.5 MPa before and 17.1 ± 3.0 MPa after a 2 h temperature treatment at 400 °C. Thus, no reduction of adhesion is revealed.

There are a variety of future research aspects. Results show that protective layers and trimming are required to achieve a stable sensor behaviour. As trimming is possible only before the deposition of protective layers, a trimming technology integrated in the sputter coating system is required. This would enable to deposit, to trim and to directly cover the sensors with a suitable coating layer. Moreover, the technology allows the stacked deposition of trimmed strain gauges, resulting in for instance multilayer strain gauge rosettes usable for detecting main stress directions. Furthermore, investigations on long-term stability have to be carried out to stabilise sensor behaviour over time and temperature. Especially, the reduction of noise alias maximum deviations and temperature limiting influences of the contacting are required. Temperature pre-treatment appears to have a positive influence on sensor behaviour and should be investigated with respect to the physical and chemical impact of time and required temperature. In addition, the identical behaviour of sensors concerning resistance change over time and temperature are of interest.

## Figures and Tables

**Table 1:** Gradient of linear regression alias *TCR* and *TCR* differences of two resistances forming one half-bridge. Measurements took place for commercial (C) and directly deposited (DD) strain gauges.

Half-Bridge	Gradient of Linear Regression alias TCR in ppm/°C		TCR Difference in ppm/°C
	R1 or R3	R2 or R4	
C HB1	28	25	3
C HB2	28	26	2
C HB3	25	24	1
DD HB1	179	182	3
DD HB2	179	180	1
DD HB3	179	183	4
DD HB4	183	183	0
DD HB5	182	183	1
